# Puerperal Eclampsia

**Published:** 1884-08-20

**Authors:** Walter H. Parcels

**Affiliations:** Lewistown, Pa.


### PUERPERAL ECLAMPSIA.

BY WALTER H. PARCELS, M.D., OF LEWISTOWN, PA.

I was called, about two o'clock on Sunday morning, April 6, 1884, to attend, in confinement, Mrs. S., aged a trifle less than seventeen years. I found the presentation normal, os uteri dilatable, head not yet engaged in the superior strait. The uterine contractions, which were mild at first, increased gradually in force and frequency; the os uteri dilated; the head descended. I mentally estimated the completion of labor by daylight. The woman was in hopeful spirits, complainining once or twice of a slight headache only; but, as the pulse was normal, or nearly so, and the face not flushed, to me the case seemed to be going along as regularly and as merrily as the chimes of a marriage bell. About five o'clock, however, she was seized with a *convulsion*, which was epileptiform in character, and came as unheralded as a lightning stroke from a clear sky. The convulsion lasted, probably, three minutes, leaving her in a semi-comatose condition. The child's head was now pressing upon the perineum, and needed only a few more pains for its delivery. Another convulsion followed a few moments later, and the woman became entirely unconscious, and all uterine contractions ceased.

The pulse became full and frequent. I immediately took about a quart of blood from the arm. Instrumental delivery at once was my opinion, as to the next step to be taken; but, as a consultation seemed desirable, I waited a little for the arrival of my friend, Dr. Sheaffer, who came within an hour, controlling the convulsions in the meantime with chloroform. The spasms recurred about every twenty minutes, the woman at no time regaining consciousness. On the arrival of Dr. S., I immediately delivered with forceps, the child being born alive, though it died thirty-eight hours afterward.

We continued the chloroform treatment for, possibly, an hour, but the convulsions persistently returned. We then used, hypodermically, about a half-grain of morphia, nearly the same dose being repeated about three-quarters of an hour later. The convulsions now became less violent and much less frequent, one interval being, at least, an hour and a half, when there was a slight *promise* of returning consciousness. As the effects of the morphia began to subside, the convulsions returned as badly as ever. About twelve hours after the morphia had been first used, nearly a grain was again hypodermically injected, the convulsions ceasing within an hour, not to return.

Whenever the patient could swallow, she was given bromide of potassium in large doses. After the cessation of the convulsions she remained in a semi-comatose condition for about thirty hours, when, awaking apparently from a sleep, she recognized her friends.

I should have added that she was kept under the influence of morphia for about twenty-four hours after the spasms had ceased to recur. The first urine that we could get was tested, and found to be, in bulk, nearly one-fourth albumen.

From this time on the case progressed well. Repeated examinations of the urine showed a gradual decrease of the albumen, which disappeared entirely in about ten days. During this time tablespoonful doses, three times a day, of Basham's mixture were substituted for the bromide.

My object in detailing this case is to call attention to the apparent superiority of the morphia treatment over the usual modes. What is the condition of affairs which gives rise to the convulsions in puerperal eclampsia? The accoucheur takes command of the obstetrical case; the way is clear; he thinks himself marching bravely on to victory, and toward new fields of conquest, when suddenly he encounters a "lion in the way." He must act at once—time is precious. Are these convulsions hysterical? Do they depend upon a sudden determination of blood to the head, congestive apoplexy? May there be a condition of uræmia, and these convulsions be the result of a toxic agent acting upon the brain? In my opinion, nearly all the very serious cases are of this last variety. How, then, shall we proceed? Venesection is certainly the first indication. In a discussion of this subject, which I heard some years ago, a physician took the ground that, as the blood in its poisoned state is the cause, we should get rid of as much of it as possible. "Yes," said another, whose sense of humor was always uppermost, "let us take it all out, *boil* it, and pump it back again."

The proposition to bleed freely is certainly based upon a sound theory. If it be true that the poisoned blood, coming in contact with the nerve centres, is the sole cause of the convulsions, is it not true that the injurious effect will be in proportion to the amount of said blood? Though "The blood is the life," we must lessen it, for it contains, in this instance, carefully concealed, the seeds of death. Venesection performed, what next? What is the pathological condition of the kidneys? Is the case one of chronic Bright's disease? It may be, and, if so, Death will probably claim his own sooner or later, no matter what our treatment may be. Are the kidneys inflamed? Possibly so; but, probably, they are only congested, as the result of pressure of the gravid uterus. The indication, then, is to remove this pressure as speedily as possible, and nature, even unaided by art, will do much toward restoring the normal condition of the kidneys. If we can, we must deliver at once. What next? Control the convulsions with some agent which will partially paralyze the nerve-centres, and wait for the urea to be eliminated from the blood. For years chloroform has been, at this stage of the case, the standard remedy for controlling the convulsions. Why rely upon chloroform? Anæsthesia, unless the inhalations are repeated, will be temporary only, and the convulsions will return. The effects of morphia will continue for a period of from eight to twelve hours. Is there a better treatment than this? Uræmia as a cause of puerperal eclampsia was first recognized only about thirty years ago. The onward march of science is irresistible. The future may produce a remedy which will act like a talisman. Will transfusion be the strong arm which will be reached forth to save these unfortunate women? Will the poisoned blood be drawn off and blood from the arm of a healthy person be substituted? Or, better yet, will a drug be discovered which, being used hypodermically, will render urea inert and harmless?—*Med. and Surg. Rep.*

